# Recent progress on the conservation physiology of birds and bats: facing a changing world on the wing

**DOI:** 10.1093/conphys/coag074

**Published:** 2026-10-13

**Authors:** Léa Lorrain-Soligon, Rodolfo O Anderson, Christine L Madliger, Nicholas C Wu

**Affiliations:** Ecology, Evolution and Development Group, Department of Ecology and Evolution, Doñana Biological Station, CSIC, Calle Américo Vespucio, 26, 41092 Seville, Spain; Department of Ecological, Plant and Animal Sciences, La Trobe University, Plenty Rd, Bundoora Victoria 3086, Australia; Department of Biology, Algoma University, 1520 Queen St. E., Sault Ste. Marie, ON P6A 2G4, Canada; Centre for Terrestrial Ecosystem Science and Sustainability, Harry Butler Institute, Murdoch University, 90 South Street, Murdoch, WA 6150, Australia; School of Environmental and Conservation Science, Murdoch University, 90 South Street, Murdoch, WA 6150, Australia

**Keywords:** Climate change, disease, habitat modification, life history, mechanisms, monitoring

## Introduction

Ecosystems are inherently dynamic, with organisms experiencing complex variation in abiotic conditions across space and time (Nilsson & Grelsson, 1995; Wingfield, 2013). Human activities are accelerating both the pace and magnitude of environmental changes, exposing wildlife to multiple, often interacting stressors (Rodgers & Gomez Isaza, 2021). Predicting how species will respond to present and future environmental changes has emerged as a central question in conservation physiology, a field aimed at understanding how environmental change influences organismal function and ultimately shapes population persistence (Wikelski & Cooke, 2006; Cooke *et al.,* 2013). Conservation physiology plays a key role in allowing researchers to assess how organisms respond to their changing environments (Fowler *et al.,* 2023), providing a common language that transcends traditional taxonomic boundaries, allowing similar conservation questions to be addressed across diverse organisms (Tomlinson, 2025). Despite the variety of environmental changes, responses are often mediated by the same physiological systems and processes. These shared mechanisms allow conservation physiology to predict species responses to change and guide effective management strategies (Wikelski & Cooke, 2006; Cooke *et al.,* 2013).

Comparative approaches across taxonomic groups can help reveal common patterns, for which birds and bats provide an excellent example. Although they are evolutionarily distinct and often active during different periods of the day, birds and bats share many ecological and physiological challenges. For example, due to their generally small body sizes, high mass-specific metabolic rates, and the energetic demands of powered flight, they are especially sensitive to thermal and hydric constraints (McNab, 2002; Speakman *et al.,* 2003; Mdluli *et al.,* 2026). Consequently, both taxa are vulnerable to rapid environmental change. Climate change is altering their thermal environments, imposing heat stress, and increasing energy and water demands (Crawford *et al.,* 2022; Freeman *et al.,* 2023; Eby *et al.,* 2024; Newman *et al.,* 2024; Coulson *et al.,* 2025). Because they often rely on specific habitats and environmental conditions to forage, reproduce, and migrate, human-driven habitat modification and anthropogenic disturbances are reshaping resource availability, physiological homeostasis, and individual health (Crawford *et al.,* 2022; Jodice *et al.,* 2022; Kaliński *et al.,* 2022; Wascher *et al.,* 2022; Alquezar *et al.,* 2023; Brodin & Watson, 2023; Harris *et al.,* 2023; DeAnglis *et al.,* 2024; Génier *et al.,* 2025; Gutierrez Ramirez *et al.,* 2025). At the same time, environmental change is influencing the susceptibility of birds and bats to pathogens, making emerging diseases an increasing conservation concern (Costantini *et al.,* 2022; Jodice *et al.,* 2022; DeAnglis *et al.,* 2024; Le Souëf *et al.,* 2024; Kwait *et al.,* 2025; McLaughlin *et al.,* 2025). Under these diverse challenges, the physiological flexibility of birds and bats, including the ability to regulate energy balance, cope with thermal stress, and maintain immune function, is a key determinant of resilience (Jodice *et al.,* 2022; Marn *et al.,* 2022; Fowler *et al.,* 2023; Gutierrez Ramirez *et al.,* 2025; Weissenfluh *et al.,* 2025).

The studies assembled in the Virtual Issue “*Facing a changing world on the wing—conservation physiology of birds and bats*” provide an opportunity to examine how common physiological mechanisms operate across a diversity of environmental challenges, and how conservation physiology can reveal the mechanisms linking environmental challenges to individual health, behaviour, and survival. They reveal responses under the challenges of disease, climate change, and anthropogenic habitat modification, as well as the physiological mechanisms underpinning resilience. Together, these themes highlight recent advances and identify future directions for the conservation physiology of birds and bats.

## Response to global change

### Climate change and thermal challenges

Climate change extends beyond an average increase in ambient temperature, including more frequent severe storms (Freeman *et al.,* 2023), and changes in humidity (Coulson *et al.,* 2025) and the thermal heterogeneity of natural habitats (Crawford *et al.,* 2022). These factors can co-vary (Freeman *et al.,* 2023; Coulson *et al.,* 2025; Mdluli *et al.,* 2026) and interact with other stressors, including disease (Newman *et al.,* 2024) and land use (Crawford *et al.,* 2022), generating both acute and chronic physiological challenges for birds and bats (Mdluli *et al.,* 2026). Acute thermal events can directly threaten survival, as illustrated by the increased risk of lethal hyperthermia under humid conditions in the trumpeter hornbill (*Bycanistes bucinator*) (Coulson *et al.,* 2025) and the substantial energetic costs associated with severe weather events in grasshopper sparrows (*Ammodramus savannarum*) (Freeman *et al.,* 2023). However, climate change also generates long-term pressures by modifying the energetic environment in which individuals forage, reproduce, and overwinter, as shown for both Arctic seabirds and bats (Eby *et al.,* 2024; Newman *et al.*, 2024). Behavioural and physiological flexibility can provide buffering capacities, but they are constrained by habitat quality and microclimatic conditions (Crawford *et al.,* 2022; Coulson *et al.,* 2025).

Collectively, the studies in the special issue reveal that predicting the impacts of climate change requires integrating physiology across scales, from individual thermal tolerance and phenotypic plasticity to demographic processes. To design effective management strategies, key knowledge gaps must be addressed, particularly regarding heat and dehydration tolerance in bats, acclimation capacity, and the scaling of physiological responses to population persistence (Mdluli *et al.,* 2026).

### Anthropogenic change, habitat modification, and resource quality

Human activity reshapes the environments birds and bats inhabit both directly, through disturbance, and indirectly, through altered resources. Papers in the special issue show that direct anthropogenic disturbance can trigger acute physiological responses, such as elevated heart rate and body temperature (Wascher *et al.,* 2022), and shifts in heterophil-to-lymphocyte ratios associated with tourist visitation (Harris *et al.,* 2023). Other studies show that chronic, low-level exposure to human activity produces more variable outcomes. For example, Alquezar *et al.* (2023) found that birds nesting near airports do not show consistently elevated corticosterone, while Brodin and Watson (2023) report that urban great tits (*Parus major*) paradoxically exhibit lower corticosterone levels than forest individuals. Likewise, Jodice *et al.* (2022) show that oil and gas activity influences baseline health parameters in coastal-nesting bird species.

Habitat modification can also alter resource quality and availability. For example, riparian and inland nesting habitats differ in the fatty acids available to aerial insectivores, with implications for nestling development (Génier *et al.,* 2025). Other studies examine the direct effects of human interventions aimed at improving resource availability or providing artificial habitats. Kaliński *et al.* (2022) show that artificial food availability shapes ketone body levels and winter energetic condition in great tits, while Crawford *et al.* (2022) demonstrate that artificial roost structures (e.g. bat boxes) can impose unintended energetic costs, with overheating risk strongly dependent on design and placement. Together, these studies show that anthropogenic pressures do not act uniformly; instead, the same driver (i.e. disturbance, urbanization, food supplementation) can raise or lower physiological stress depending on species, context, and exposure history. A key gap is the scarcity of longitudinal or cross-species comparisons that would allow us to better predict how taxa are affected by human-modified landscapes and associated disturbances.

### Health, immunity, and disease

Global change is also exposing bird and bat populations to novel physiological challenges that affect health across scales, from individual immune responses to emerging disease outbreaks. Conservation physiology can again provide a useful approach by revealing both the mechanisms and consequences of disease vulnerability in a changing world. Establishing robust health benchmarks is fundamental to detecting change. Eleven-year monitoring of the endangered Carnaby’s cockatoo (*Zanda latirostris*) nestlings by Le Souëf *et al.* (2024) provides valuable physiological baseline data across time, documenting age-specific haematological and biochemical reference ranges while revealing low but persistent exposure to pathogens, including beak and feather disease virus, avian polyomavirus, and Chlamydia. Such datasets are increasingly important as habitat loss, climatic extremes, and other anthropogenic stressors have the potential to alter host condition and disease susceptibility (Mahon *et al.,* 2024).

Surveillance and early detection of impacts on health are also crucial. Jodice *et al.* (2022) demonstrated how physiological indicators in the eastern brown pelican (*Pelecanus occidentalis*) can be incorporated into wildlife health assessments to evaluate population condition and emerging threats before overt demographic impacts become apparent. At larger spatial scales, McLaughlin *et al.* (2025) showed that maternal antibodies in seabird eggs provide a powerful and minimally invasive tool for tracking exposure to highly pathogenic avian influenza. Widespread increases in anti-H5 antibodies following the H5N1 outbreaks of 2022 indicate substantial variation among species in exposure, survival, and immune response, emphasizing that disease risk is unlikely to be distributed evenly across communities.

Moving beyond surveillance towards mechanisms, Costantini *et al.* (2022) and DeAnglis *et al.* (2024), Kwait *et al.* (2025) provide mechanistic insights into how pathogens and environmental conditions can influence host physiology. Their findings reveal that disease outcomes are shaped not only by pathogen presence but also by the nature of the host immune response to anthropogenic stressors such as habitat fragmentation (DeAnglis *et al.,* 2024). In Egyptian fruit bats (*Rousettus aegyptiacus*), experimental bacterial sickness induced increases in haptoglobin and lysozyme, but did not alter markers of oxidative damage or antioxidant capacity, highlighting the complex relationship between immune activation, inflammation, and oxidative physiology (Costantini *et al.,* 2022). In little brown bats (*Myotis lucifugus*) affected by white-nose syndrome, transcriptomic evidence points to immune dysregulation, heightened inflammatory signalling, and the potential development of cytokine storm-like responses and sepsis (Kwait *et al.,* 2025). Together, these studies demonstrate that understanding resilience to global change requires attention not only to pathogens themselves, but also to the physiological processes that mediate host responses to infection, recovery, and survival.

### Life history decisions and fitness outcomes

Faced with environmental challenges, birds and bats may also alter life history decisions, resulting in consequences for reproductive success and survival. Papers in the special issue illustrate that physiological metrics can provide information on the mechanisms underlying the life history decisions of birds related to migration, nesting initiation and egg laying, and foraging during the reproductive season. For example, Weissenfluh *et al.* (2025) showed that Franklin’s gulls (*Leucophaeus pipixcan*) that initiated nesting later in the breeding season had lower size-corrected body mass, reduced immune function, and higher maximum corticosterone concentrations after an acute stressor. Put simply, the timing of nesting in this species is related to physiological condition, providing insight into how resource availability may translate into population-level shifts in nesting phenology. Physiological information also has a role in this context when employing modelling techniques. Using a Dynamic Energy Budget model, Marn *et al.* (2022) successfully predicted the reproductive ontogeny of bobwhite quail (*Colinus virginianus*), including egg size and egg-laying rate. Such models can be expanded to study how changing anthropogenic pressures influence the energy-related physiological systems that underpin reproductive decisions. Indeed, research by Eby *et al.* (2024) indicates that when sea ice concentrations are high during the incubation period, thick-billed murres (*Uria lomvia*) that make longer, deeper dives are in a better nutritional state. These results are important for interpreting how changing extrinsic conditions influence the timing and ability of birds to make flexible foraging decisions during the breeding season.

Physiology is also informative for understanding migration dynamics. With quantitative magnetic resonance measurements of fat and lean mass, Gutierrez Ramirez *et al.* (2025) documented that migratory songbirds traversing the eastern Gulf of Mexico are arriving in lower condition than previously reported, indicating that many species are pushing the physiological limits of energy management. Importantly, the increase in body mass that occurs during stopover in this region is characterized by rapid lean mass accumulation, drawing attention to the importance of stopover habitats for providing not just fat resources, but water, sleep, and access to protein. Other investigations can help to determine which physiological metrics may be most useful for predicting migration movements. Fowler *et al.* (2023) investigated whether oxidative physiology is related to individual migration metrics including maximum distance travelled, migration distance, and migration rate, in long-tailed jaegers (*Stercorarius longicaudus*) and Arctic terns (*Sterna paradisaea*). No relationship between individual oxidative variables and migration metrics emerged. However, haematocrit, a metric of aerobic capacity, was positively correlated with rate of travel for Arctic terns. Drawing attention to the context in which different physiological variables are useful for understanding animal movement is integral to their future application in both experimental and applied settings. Collectively, as part of the special issue, these articles are contributing to an outstanding challenge in the application of physiological tools to conservation science: investigating the capacity to scale individual measures of physiology to population-level changes and consequences.

## Discussion

Climate change, habitat modification, anthropogenic disturbance, and disease differ in their immediate causes, yet they all ultimately influence energy balance, thermoregulation, immune function, stress physiology, and behaviour. Accordingly, the studies presented highlight a diversity of physiological tools and approaches, encompassing cellular damage and oxidative physiology (Costantini *et al.,* 2022; Fowler *et al.,* 2023; Mdluli *et al.,* 2026), haematology and immune function (Costantini *et al.,* 2022; Jodice *et al.,* 2022; Freeman *et al.,* 2023; Harris *et al.,* 2023; DeAnglis *et al.,* 2024; Le Souëf *et al.,* 2024; McLaughlin *et al.,* 2025; Weissenfluh *et al.,* 2025), energetic and nutritional state (body mass, energetic stores, ketone bodies, nutritional biomarkers; Kaliński *et al.,* 2022; Freeman *et al.,* 2023; Eby *et al.,* 2024; Génier *et al.,* 2025; Gutierrez Ramirez *et al.,* 2025; Mdluli *et al.,* 2026), as well as trophic level (Harris *et al.,* 2023), glucocorticoid physiology (Alquezar *et al.,* 2023; Brodin & Watson, 2023; Weissenfluh *et al.,* 2025), energy balance and metabolism (Wascher *et al.,* 2022; Mdluli *et al.,* 2026), aerobic capacity (Fowler *et al.,* 2023), foraging behaviours and activity budgets (Crawford *et al.,* 2022; Eby *et al.,* 2024), evaporative water loss (Mdluli *et al.,* 2026), thermoregulation (Wascher *et al.,* 2022), torpor patterns (Crawford *et al.,* 2022; Newman *et al.,* 2024), and mortality (Coulson *et al.,* 2025; Mdluli *et al.,* 2026). The studies in the issue also highlight physiological flexibility as a key determinant of resilience. Behavioural and physiological adjustments, including behavioural thermoregulation, torpor use, and foraging flexibility, can buffer organisms against environmental stressors and reduce immediate mortality risk (Eby *et al.,* 2024; Mdluli *et al.,* 2026). However, these responses often come at sublethal costs by reducing opportunities for foraging, predator avoidance, or reproductive investment (Mdluli *et al.,* 2026). These patterns highlight the importance of distinguishing acute responses from chronic physiological adjustments, whose cumulative effects are likely to have important consequences for population persistence, as highlighted in the context of thermal biology (Mdluli *et al.,* 2026). Understanding these relationships, which are poorly investigated, particularly in bats, will require greater integration of physiological measurements with long-term population monitoring, while accounting for other stressors. Given that environmental drivers rarely operate in isolation, greater attention should be given to phenotypic plasticity, developmental effects, and the interaction between multiple stressors.

Responses differ, however, among species, populations, and life-history stages according to ecological traits, habitat characteristics, and environmental conditions. For example, thermal vulnerability depends on climate but also on body size, roosting or nesting ecology, and thermoregulatory strategy (Crawford *et al.,* 2022; Mdluli *et al.,* 2026). Similarly, responses to anthropogenic disturbance vary considerably among bird species (Alquezar *et al.,* 2023), while intrinsic factors such as sex and reproductive demand influence foraging behaviour and energetic state (Eby *et al.,* 2024). Unexpectedly, urban great tits showed lower feather corticosterone than forest birds, further highlighting the importance of considering social, intrinsic, and environmental contexts when interpreting differences (Brodin & Watson, 2023). Identifying the ecological and physiological traits that determine vulnerability should become a priority for future comparative studies. Such comparisons are currently constrained by taxonomic and geographic biases, particularly for bats, for which physiological responses remain substantially less studied than in birds, especially in terms of relating physiological variation to fitness (Mdluli *et al.,* 2026). This asymmetry is not specific to this issue, as bats remain understudied relative to birds in climate change research more broadly (Festa *et al.,* 2023). Closing this gap should be a priority for future conservation physiology research.

Such context dependence also highlights a more fundamental challenge for conservation physiology: we need to establish robust physiological baselines across species, populations, and life-history stages. In the special issue, baseline haematology and blood biochemistry have been established for pelicans (Jodice *et al.,* 2022) and a threatened cockatoo (Le Souëf *et al.,* 2024), and Fowler *et al.* (2023) established baselines for multiple oxidative physiology metrics and aerobic capacity in two seabird species. Importantly, physiological measurements may also provide a means of linking individual condition to population-level responses (Weissenfluh *et al.,* 2025). Repeated physiological measurements across key life-history stages could improve our ability to translate individual responses to environmental change into predictions of population consequences. Studies in this issue also demonstrate minimally invasive tools for tracking physiological state (Gutierrez Ramirez *et al.,* 2025; McLaughlin *et al.,* 2025), offering an approach for scaling physiological monitoring across more species and populations. Fowler *et al.* (2023) drew attention to the importance of determining how multiple physiological measurements are integrated (e.g. as components of a biological system with shared functionality) and/or inter-related. Such investigations will allow researchers and practitioners to effectively build physiological panels that more holistically assess stress, energetic demand, and tolerance. Nutritional baselines are rarer still. Studies on lean mass and ketone bodies (Kaliński *et al.,* 2022; Gutierrez Ramirez *et al.,* 2025) emphasize this gap. Without reference ranges, it is hard to judge whether a value signals good or poor condition. Integrating physiological measurements with long-term monitoring and predictive modelling (particularly biophysical modelling) will considerably strengthen evidence-based conservation planning (Marn *et al.,* 2022; Coulson *et al.,* 2025; Mdluli *et al.,* 2026), allowing quantification of the physiological processes underlying vulnerability and resilience. Importantly, birds and bats also face shared physical threats not covered in depth here. These include collision with infrastructure, light pollution, exposure to pollutants, and pesticides, and the high energetic demands of flight (O’Shea *et al.,* 2016; Guigueno *et al.,* 2019; Frick *et al.,* 2020; Marra *et al.,* 2026).

## Conflicts of interest

The authors declare no conflict of interest.
